# Assessing the effect of roads on mountain plant diversity beyond species richness

**DOI:** 10.3389/fpls.2022.985673

**Published:** 2022-09-26

**Authors:** Honglin Li, Peng Luo, Hao Yang, Chuan Luo, Wenwen Xie, Honghong Jia, Yue Cheng, Yu Huang

**Affiliations:** ^1^ CAS Key Laboratory of Mountain Ecological Restoration and Bioresource Utilization & Ecological Restoration and Biodiversity Conservation Key Laboratory of Sichuan Province, Chengdu Institute of Biology, Chinese Academy of Sciences, Chengdu, China; ^2^ College of Life Sciences, University of Chinese Academy of Sciences, Beijing, China

**Keywords:** hill numbers, roads, mountain plant assemblages, functional traits, phylogenetic diversity, biodiversity hotspots

## Abstract

A comprehensive understanding of the effects of mountain roads on plant diversity is critical to finding the most effective solutions for managing this particular driver. Little is known, however, about the simultaneous effects that road have on the multiple facets of biodiversity, although roads are considered to be one of the major disturbances in the Qionglai mountain range. In this study, we analyzed the impact of roads on the multiple facets of plant diversity (taxonomic, functional and phylogenetic diversity) in the study area using Hill numbers by comparing plant diversity between roadside and interior plots at the landscape scale, then, we used linear mixed models to analyze the effect of mountain roads on the multiple facets of plant diversity along an elevational gradient. The results showed that the roadside plots lacked 29.45% of the total number of species with particular functional traits (such as a relatively high specific leaf area (SLA), a relatively low leaf dry matter content (LDMC) and relatively old clades) and exclusively contained 14.62% of the total number of species. Compared with the interior community, the taxonomic, functional and phylogenetic diversity of roadside community decreased by no more than 26.78%, 24.90% and 16.62%, respectively. Taxonomic and functional diversity of dominant and common species showed greater changes to road disturbances, while rare species showed the greatest change in phylogenetic diversity. Taxonomic homogenization of roadside communities was accompanied by functional and phylogenetic homogenization. Additionally, the impact of roads on these three facets of plant diversity showed the characteristics of peak clipping along the elevation gradient. Our findings highlight the negative impact of roads on the taxonomic, functional and phylogenetic diversity of the Qionglai mountain range, as roads promote communities that are more similar in taxonomic, functional, and phylogenetic composition, and to a greater extent contributed to compositional evenness. These effects tend to be functionally and phylogenetically non-random, and species in some clades or with some functional traits are at higher risk of loss. Our results are important for the conservation and management of nature reserves, especially for local governments aiming to create new infrastructure to connect natural mountainous areas.

## Introduction

Although covering a relatively small fraction of Earth’s area (13%~25%, Ives, 1997), mountainous regions support a significant one-third of all terrestrial biological diversity due to their topographic and environmental complexity (Tang et al., 2006; Perrigo et al., 2020; Brighenti et al., 2021). A common feature of many mountains is that they contain road networks that connect lowlands with high-elevation sites. Economic development will mandate the construction of many new roads in the coming decades (Seto et al., 2012; Xie et al., 2021), and these roads will extend into once-inaccessible mountainous areas; this will cause these regions to face trade-offs between biodiversity conservation and economic growth (Miller et al., 2021). Mountain roads can drive various changes in the global mountain community composition and structure *via* habitat losses (Laurance et al., 2009), fragmentation (Delgado et al., 2007) and pollution from combustion of fossil fuels by the on-road vehicles (Karmakar et al., 2021), all of which affect biodiversity and impact the structure and functioning of ecosystems (Millennium Ecosystem Assessment, 2005; De Pauw et al., 2021).

During the past two decades, research into the effects of roads on plant diversity in mountainous regions has gained momentum (Pauchard and Alaback, 2004; Barbosa et al., 2010; Seipel et al., 2012). Previous studies have focused on the impact of roads on plant communities by assisting the dispersal of non-native species or native generalists, and the results suggest that mountain roads and the colonization of generalists lead to taxonomic homogenization of plant community (Arévalo et al., 2010; Haider et al., 2018). Most of what we currently know about the influence of roads on plant diversity across the globe has come from studies of plant species richness and composition (Lembrechts et al., 2017; Dar et al., 2018; Rashid et al., 2021). However, no studies have investigated how the functional traits and phylogenetic clades respond to environmental filtering and biological competition induced by mountain roads, thus limiting our ability to devise practical strategies for conserving plant diversity.

The Qionglai mountain range, located on the easternmost edge of the Hengduan Mountains in Sichuan Province, is home to many temperate vascular plant species, some of which are ancient relic and endemic species (Myers et al., 2000; Boufford, 2014). This range was recognized as a World Heritage Site in 2006 due to its unique and valuable plant diversity (Boufford, 2014). Climate change and anthropogenic disturbances are identified as the two major reasons driving recent changes in plant diversity and community composition in this region (Li et al., 2013; Qiu et al., 2014; Salick et al., 2019; Yin et al., 2020). Conservation efforts have focused on managing the human activities that affect plant diversity (Kang, 2021; State Council Information Office of China (SCIO), 2015). With the official establishment of the Giant Panda National Park (GPNP) in 2020, clear policies and guidelines for human activities such as enforcement, tourism and infrastructure development guidelines are needed within this region. A comprehensive study of the impact of roads on the plant diversity would provide managers with enough information for them to better predict the gains and losses associated with various decisions when planning new roads or closing existing roads.

Roads are gathering points of human activities, carrying material and cultural exchanges between lowlands and high-elevation sites. In this study area, Yin et al. (2020) found that road was one of the major human activities by quantifying the relative contributions of climate change and human activities to net primary productivity and Kang (2021) concluded by reviewing the impact of anthropogenic disturbance on the habitat of giant pandas that roads were the major factor affecting the habitat of giant pandas. In addition, the State Forestry Administration also believed that roads are a major human disturbance in the study area (State Forestry Administration (SFA), 2015). Nonetheless, it is unclear whether functional and phylogenetic diversity change as a result of road disturbances, whether such changes are in the same direction as taxonomic diversity (i.e., decreased diversity) and, if so, with what intensity these changes occur.

Moreover, biodiversity includes not only the number of species but also aspects such as identity, dominance, evenness and rarity (Hill et al., 2016; Hillebrand et al., 2018). Thus, measuring plant species richness alone may not be sufficient to represent changes in plant diversity (Berdugo et al., 2019). Within communities, species composition changes may have effects on ecosystem functions that are equal to, or stronger than, the effects of changes in species richness (Shi et al., 2016). Thus, assessing road-driven shifts in species abundance is paramount for understanding how these compositional changes affect community and ecosystem functioning in response to human disturbances.

In this study, we investigated whether and how mountain roads affected the taxonomic, functional and phylogenetic diversity of plant communities in the Qionglai mountain range, and whether the responses of plant communities to roads are dependent on the location of the plant communities on the elevation gradient. Specifically, we aimed to answer the following questions: (i) Have there been changes in the three facets of plant diversity due to mountain roads by comparing the pattern of species diversity between roadside plots and interior plots, and if so, to what extent? (ii) Are the shifts in plant diversity synchronous and monodirectional; i.e., does plant diversity uniformly increase or decline along the elevation gradient? For the first question, we expect that species conserved from interior plots or spread along roads may be closely related (even sister species) or functionally similar species. Therefore, roads lead to decreases in overall plant diversity indices by inducing changes in species richness and abundance (Haddad et al., 2015; Razafindratsima et al., 2018). Furthermore, we predict that changes in functional and phylogenetic diversity will be smaller than changes in taxonomic diversity due to functional and phylogenetic redundancy. In relation to our second question, we hypothesize that the pattern of the effects of roads on the functional and phylogenetic diversity indices would be consistent with the effect on taxonomic diversity, i.e., the effects would vary with the elevation gradient. Based on our previous results (Li et al., 2022), the expected negative impacts of roads on diversity may be greater at moderate and high elevations than at low elevations. Answering the abovementioned questions and filling this knowledge gap are important, as changes in the three facets of plant diversity may have different implications for the functioning of ecosystems and their responses to human activities.

## Methods

### Study area

**Figure 1:**
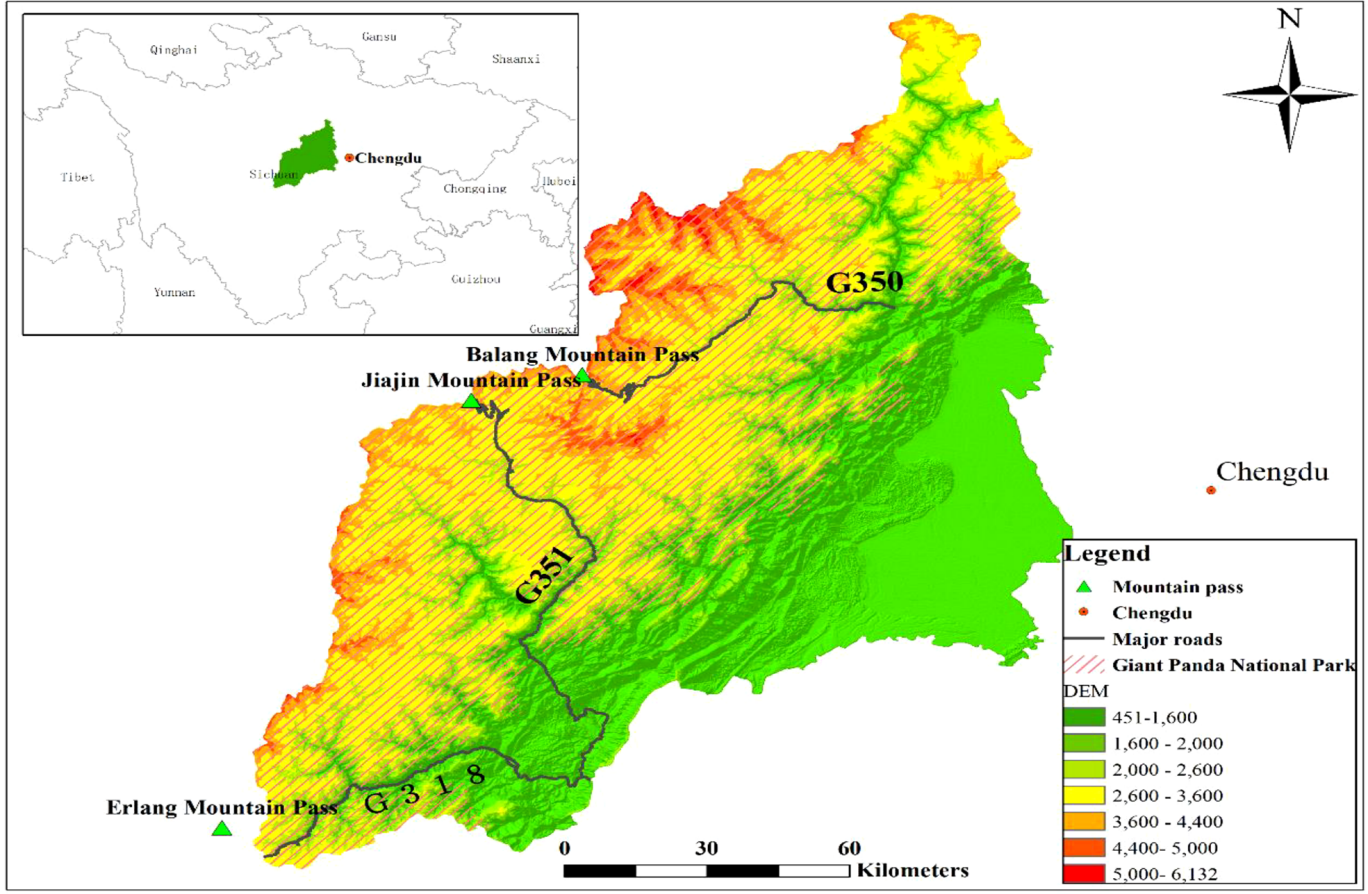
Location map of the study area.

The Qionglai mountain range is located in the upper Yangtze River of northwest Sichuan Province (**Figure 1**), China (102°16′ E-104°10′ E, 29°49′ N-31°31′ N). The area comprises a major portion of Giant Panda National Park. The mountain range is situated in the transitional zone between a subtropical monsoon climate zone and a continental plateau climate zone. Its highest peak, Siguniang Mountain, has an elevation of approximately 6250 m a.s.l. The landscape along the elevation gradient is dominated by arid shrubs, subtropical evergreen and deciduous broadleaf forests, coniferous forests, subalpine coniferous forests, scrub meadows, alpine talus vegetation, and permanent snow belts (Sichuan Flora Editorial Committee, 1981). Of these, arid shrubs and permanent snow belts were not included in this study (Li et al., 2022).

G350, G318, G213 and G351 are four major roads in the Qionglai mountain range (Xu et al., 2006; Li et al., 2022). The total length of major roads in the mountain range is about 799.84 km, and the road density is about 0.05 km/km^2^. Only part of three major roads, G350, G318 and G351, are located in the national park (**Figure 1**) and these road segments were selected for this study. The density of major roads in the national park is about 0.02 km/km^2^. All road segments selected in this study comply with the third-level highway technical standards in the Technical Standards of Highway Engineering (JTG B01-2014) issued by the Ministry of Transport of China. These road segments have been in place for more than 50 years, with an average daily traffic volume of more than 1,500 vehicles. Other roads in the area are low-traffic country trails. That is to say, the selected road segments have the same properties, and they collectively represent the overall road conditions within the national park in the Qionglai mountain range.

### Floristic data

**Figure 2:**
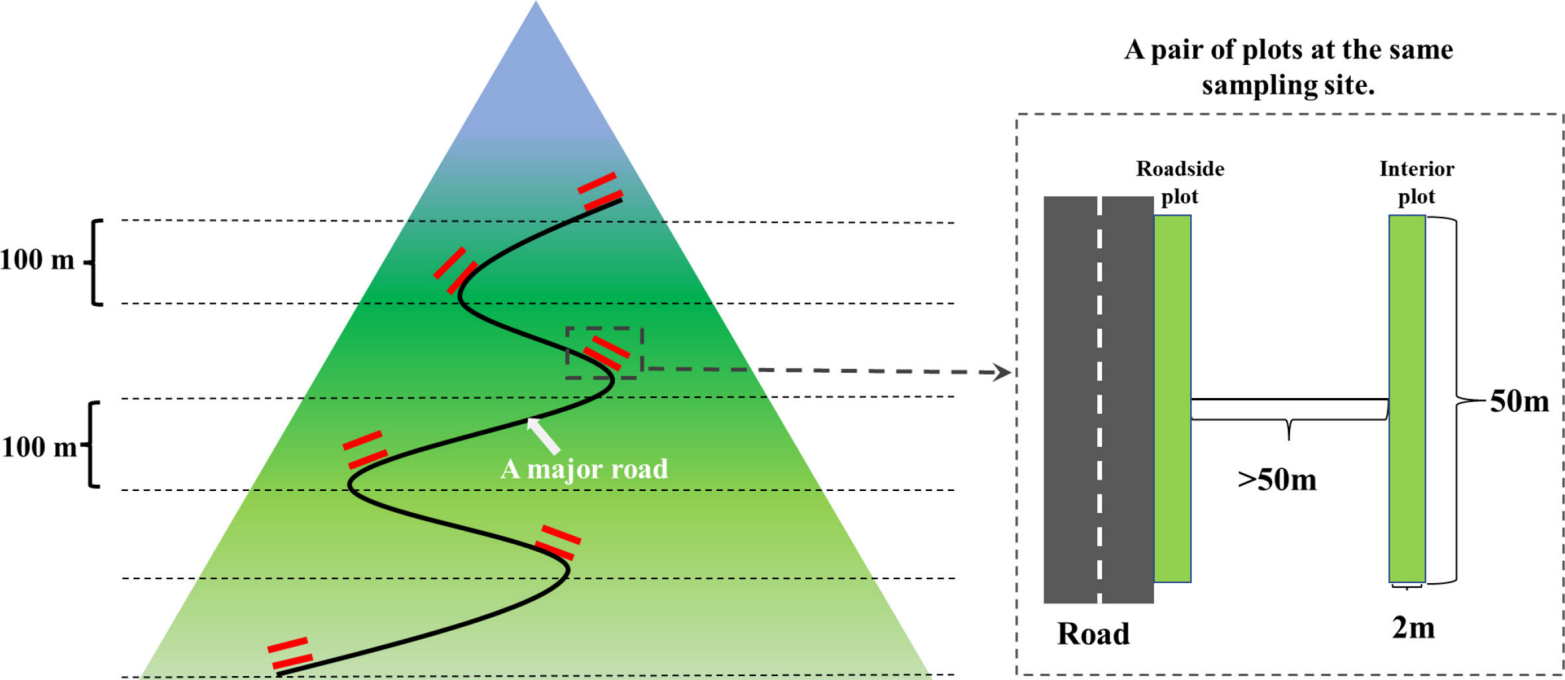
Schematic diagram of sampling site and plot setting.Data analysis.

In this work, field surveys were conducted along the selected segments of three national roads (G318, G350 and G351) in the Qionglai mountain range. For each of the three roads, the lowest and highest sampling sites were first determined. The lowest sampling site for each road was the location that can be sampled with the greatest avoidance of interference from other human activities. The highest sampling site of each road was more than 1,000 meters from the tunnel entrance or high passes. The sampling range of this study includes four vegetation types. To ensure adequate replicate sampling for each vegetation type, referring to the research by Zhang et al. (2009), a sampling site was systematically located at 100 m a.s.l. elevation intervals from the lowest sample site to the highest sample site (see **Figure 2**). In order to avoid interference from other human activities to the greatest extent, all selected sampling plots were required to be free of farmland or people gathering points within 1,000 m. In addition, sampling sites avoided pasture paths and sites with large amounts of livestock manure. In order to meet the sampling conditions described above, coupled with the difficulties of topography and access, 38 sampling sites were finally established in this study [see also Li et al. (2022)]. Four vegetation types including subtropical evergreen and deciduous broadleaf forests, coniferous forests, subalpine coniferous forests, scrub meadows included 8, 6, 15 and 9 sampling sites, respectively.

At each sampling site, two 2 m*50 m plots parallel to the road were established, which were called a pair of plots (interior and roadside plots) (see **Figure 2**). One plot was parallel and next to the roadside was collectively referred to as the roadside plot (representing the communities affected by road). According to the previous study results on the impact of roads on adjacent ecosystems (Hansen and Clevenger, 2005; Arévalo et al., 2010; Deljouei et al., 2018), and considering the accessibility of plots during sampling, another one plot was placed parallel to the roadside plot and was more than 50 m away from the roadside (representing the natural communities away from the road). In the study, we established a total of 38 plot pairs and 76 plots. We recorded the species richness of vascular plants and the abundance of each species in each plot. In the field, we taxonomically identified the vascular plants based on the Flora of China (http://www.eFloras.org). Take photos and collect specimens of uncertain or unknown plants and bring them back to the laboratory to continue searching for identification information. Standardized nomenclature for all species according to the Plant List (http://www.theplantlist.org/).

### Functional traits

For each of the 978 species in the 76 plots, we recorded 12 functional traits (**Table S3**). These heterogeneous characteristics collectively comprise the functional facet of plant diversity required for the calculation of Hill numbers. Data on 3 quantitative functional traits, specific leaf area (SLA), leaf dry matter content (LDMC), and leaf thickness (Lth), were obtained from field measurements. The SLA was calculated as the ratio of the fresh leaf area to the leaf dry matter content. An HPG3110 scanner (Yaxin-1241, Beijing Yaxin Liyi Technology Co., Ltd., Beijing) was used to measure the fresh leaf area. The LDMC was obtained by storing the collected leaves in silica gel and taking them back to the indoor laboratory, and then weighing the leaves after oven-drying at 60°C for at least 48 hours. The Lth was obtained by measuring the thickness of fresh leaves with Digital Vernier caliper with 0.01 mm accuracy (PD-151, Prokit’s Industries Co., Ltd., China). All traits were measured on five to 30 healthy mature individual plants, depending on the trait and species, following Pérez-Harguindeguy et al. (2013). The data of the remaining 9 qualitative functional traits were obtained through the TRY database (Kattge et al., 2020; www.try-db.org) and Flora of China (English edition; www.efloras.org). A functional distance matrix was then built based on the calculation of the Gower distance (Gower, 1971) to allow for the mixing of categorical, ordinal, and continuous data.

To analyze the specific functional compositions of the roadside and interior communities, we first used the *functcomp* function of the R package FD (Laliberté and Legendre, 2010) to calculate the community-weighted means (hereafter referred to as CWMs) and non-weighted means (hereafter referred to as CMs) of all functional traits based on the abundances of species in each community. Nonmetric multidimensional scaling (NMDS) based on these community-type-level CWM and CM values of the 76 plots was performed using the *metaMDS* function in the vegan package (Oksanen et al., 2020) to identify the functional determine differences among all plots.

#### Phylogenetic tree construction

To create a phylogenetic tree for each plot type, we pruned our species list from the mega-tree GBOTB.extended.tre, which currently represents the largest available phylogenetic treatment of vascular plants (Zanne et al., 2014; Smith and Brown, 2018). This was done by using the V.PhyloMaker package with the *phylo.maker* function and using scenario 3 method to add plant species to the phylogenetic tree (Jin and Qian, 2019).

#### Taxonomic, functional and phylogenetic facets of plant diversity

The three plant diversity facets were converted to Hill numbers expressed as the effective numbers of species (Jost, 2007; Chao et al., 2014; Chiu et al., 2014). We calculated all plant diversity index facets at the plot level (n = 38 for each plot type), using the “hillR” package (Li, 2018). Then, we examined the pairwise correlations of the multifaceted diversity indices using Pearson’s correlation coefficient.

When q = 0, the diversity index equals the species richness, giving disproportionate weight to rare plants (Jost, 2006; Tuomisto, 2010; Arroyo-Rodríguez et al., 2013; Vega-Álvarez et al., 2019). When q = 1, the diversity index is equivalent to Shannon’s diversity index, and each species is weighted by its abundance in the community (Jost, 2007). Finally, for q = 2 (representing the inverse Simpson concentration), dominant species are favored while rare species are discounted (Jost, 2010; Chao et al., 2012). The functional and phylogenetic counterparts of taxonomic diversity for q = [0, 1, 2] are presented in Chao et al. (2014).

### Statistical analysis

To explore the overall compositional differences between the roadside and interior communities, we also evaluated the taxonomic, functional and phylogenetic dissimilarities using normalized dissimilarity measures based on the Hill numbers derived (Chao et al., 2014; Chiu et al., 2014) at the local (i.e., comparing each individual plot to other plots surveyed within the same plot type) and regional (i.e., comparing each individual plot to the pool of plots surveyed within the same plot type) scales. Then, we used linear mixed models to analyze the effects of mountain roads on three facets of plant diversity (taxonomic, functional and phylogenetic diversity) for Hill numbers q = 0, q = 1, and q = 2 along the elevation gradient. The plot type (a factor with two levels: roadside vs. interior plot), elevation and its quadratic term, and interaction between the covariate and factor were used as fixed terms in the models. Due to the nested structure of the data, linear mixed models with the site (38 levels corresponding to 38 roadside and interior plot pairs) set as a random effect (random intercept) nested within roads (three levels corresponding to three roads) were used (thus, 76 plots were nested in 38 sites nested in three roads) (Li et al., 2022). We built all possible fixed factor models and performed model selection based on the Akaike information criterion corrected for small sample sizes (AICc). We tested all possible models with the *dredge* function from the package MuMIn (Bartoń, 2022). The mixed effects models were fitted with the function *lme* in the nlme package (Pinheiro et al., 2021).

To reduce the model selection bias effects on the regression coefficient estimates in these selected subsets (Burnham & Anderson, 2002), we calculated the linear mixed model estimate values and their standard errors (SEs) for the entire set of plausible models (ΔAICc< 2) and for marginal( $R_{m}^{2}$ ) and conditional ( $R_{c}^{2}$ ) R^2^ values. Following the methodology proposed by Nakagawa and Schielzeth (2013), $R_{m}^{2}$ values accounted for the proportion of variance explained by the fixed factors of the model, while the $R_{c}^{2}$ values yielded the proportion of variance explained by the fixed and random model factors. All statistical analyses were run in the statistical program R 4.1.2 (R Core Team, 2021; https://www.r-project.org/).

## Results

### Three facets of plant diversity

Overall, 978 species belonging to 395 genera and 113 families were collected in this study. We found 177 species whose abundances reached 0.1% of the total abundance (**Table S1**) and 98 species whose abundances represented less than 0.001% of the total abundance (**Table S2**). In roadside plots, 689 plant species (70.45%) were found, in contrast to the 835 species (85.38%) found growing in interior communities. The 143 species (14.62%) occurring exclusively in roadside communities belonged to 14 families of angiosperms and 1 family of gymnosperms. Plants uniquely occurring interior plots included 288 species (29.44%), 70 genera and 40 families (10 families of pteridophytes, 29 families of angiosperms and 1 gymnosperm family). Comparisons of the different facets of plant diversity revealed that lower taxonomic and functional diversity index values were obtained for all q orders (q= [0, 1, 2]) compared to the phylogenetic diversity values (**Table 1**).

**Table 1:** Descriptive statistics of taxonomic, functional and phylogenetic Hill numbers for q = [0, 1, 2].

	Plot type	The value of q	Taxonomic Hill number	Functional Hill number	Phylogenetic Hill number
**Mean ± SD**	**Roadside plot**	0	66.95 ± 14.52	73.30 ± 16.73	4545.11 ± 698.65
		1	22.87 ± 8.37	26.48 ± 9.00	862.60 ± 78.87
		2	13.34 ± 5.81	16.03 ± 6.00	554.34 ± 17.55
	**Interior plot**	0	77.84 ± 17.90	85.75 ± 22.71	5522.17 ± 762.10
		1	31.04 ± 7.32	35.00 ± 7.91	1034.26 ± 125.95
		2	19.09 ± 5.48	21.85 ± 5.47	605.62 ± 70.76
**Range**	**Roadside plot**	0	43-103	47.27-112.96	3212.00-5846.13
		1	10.24-50.08	14.11-56.68	654.64-1077.14
		2	5.00-32.64	7.44-37.91	498.46-591.18
	**Interior plot**	0	47-119	48.30-144.03	4375.04-7221.59
		1	18.70-44.21	18.79-50.35	828.62-1423.17
		2	9.73-30.58	12.06-31.77	549.89-918.44
**Relative change rate in diversity indices**		0	-12.38%	-13.30%	-16.62%
		1	-24.25%	-23.40%	-15.51%
		2	-26.78%	-24.90%	-7.54%

And rates of change in diversity indices for roadside communities relative to interior plots.

In addition, the three facets of plant diversity showed the same arrangement from relatively high to low values in the two categories of plant plots: interior plots > roadside plots (**Figure S1**). The taxonomic and functional diversity levels showed the strongest responses in dominant species, whereas phylogenetic diversity showed the strongest effects in rare species (**Table 1**). The Pearson correlation results derived for the overall plant diversity indices showed that the correlation coefficient between the taxonomic level (^q^TD), functional level (^q^FD) and phylogenetic level (^q^PD) declined with increasing q-values (**Figure S2**). Positive correlations were obtained between the taxonomic level (^q^TD) and functional level (^q^FD) (ρ > 0.963). The strongest correlations (ρ > 0.988) were found between the taxonomic level and functional level for q = 0 (^0^TD vs. ^0^FD) in the interior community, whereas the weakest relationship was found between the functional diversity index at q = 0 and the phylogenetic diversity index at q = 2 in the roadside plots (^0^FD vs. ^2^PD, ρ = 0.46).

### NMDS analyses of CWM and CM

**Figure 3:**
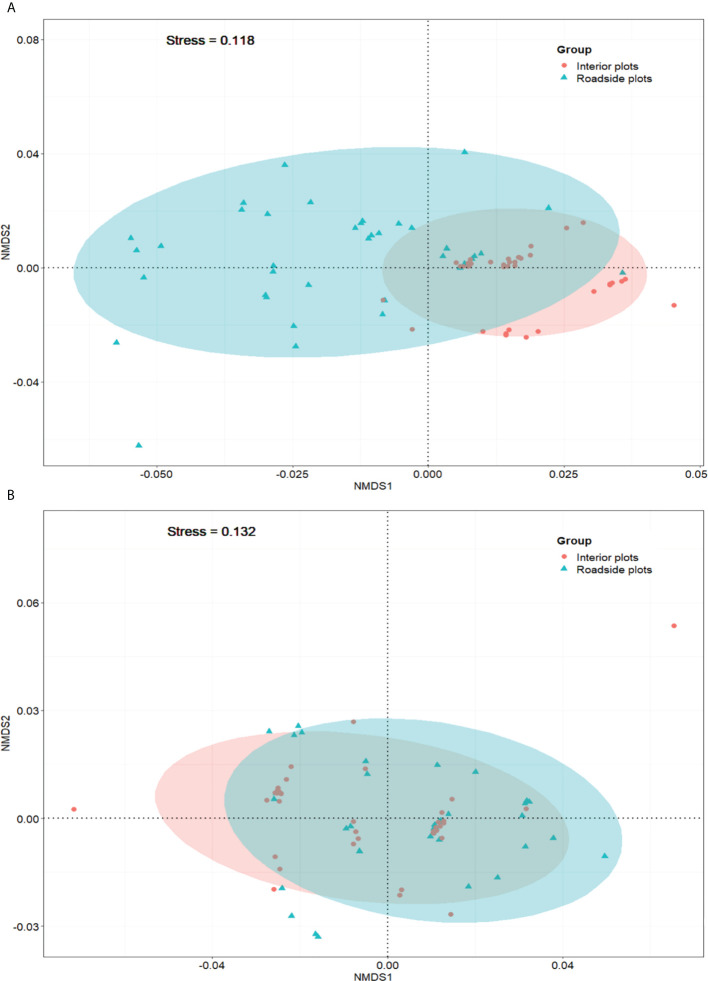
NMDS of 76 plots based on their CWM **(A)** and CM **(B)** values.

According to the NMDS, the functional identity patterns were dissimilar between roadside and interior plots (**Figure 3**). The stress values of the NMDS analyses were 0.118 and 0.132 (<0.2) for CM and CWM, respectively, indicating a good representation of data in the two-dimensional NMDS graphs. The NMDS diagram of CM showed that a clear difference in functional compositions between the roadside and interior plots (**Figure 3A**). Regarding the CWMs, roadside communities were dominated by species with fast leaf economics (such as relatively high SLA and low LDMC values), and interior communities were dominated by species with slow leaf economics, although there was no clear discrimination between roadside and interior plots in the NMDS diagram (**Figure 3B**).

### Compositional differences between the roadside and interior vegetation communities

**Figure 4:**
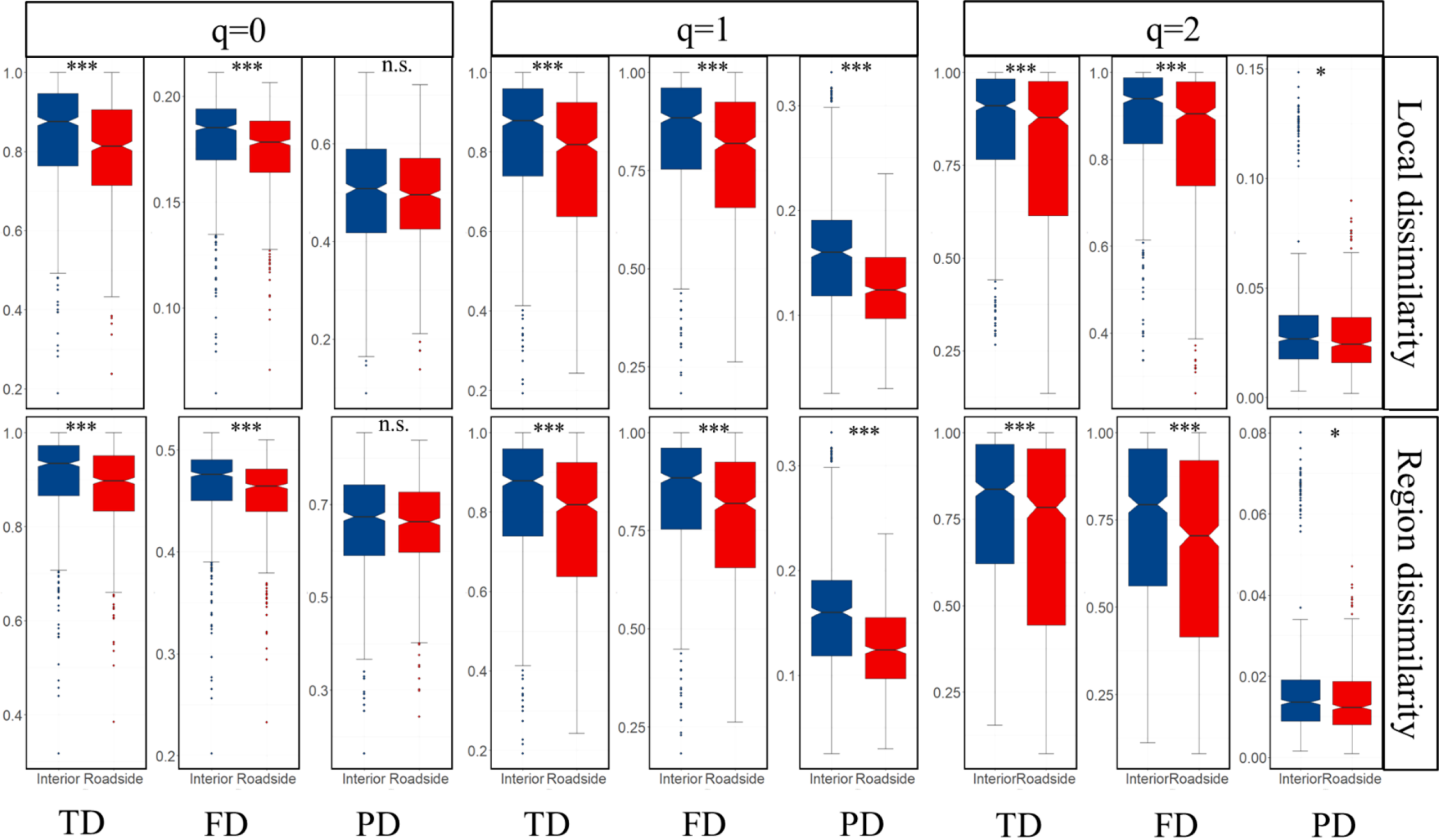
Boxplot comparing mean dissimilarity measures for interior and roadside plots for taxonomic [^q^TD], functional [^q^FD] and phylogenetic [^q^PD]) diversity for q = [0, 1, 2]. Error bars represent 95% confidence intervals. ***P < 0.001, *P < 0.05, n.s. not significant difference between roadside and interior communities.

Both the local and regional mean dissimilarity values were lower in the roadside communities than in the interior communities for all diversity indices and q orders. The greatest differences were observed for the taxonomic and functional diversity levels at q=0 at the regional scale and at q= 2 at the local scale, whereas the lowest differences were found for phylogenetic dissimilarity at q=2 at both scales (**Figure 4**). In general, roads increased the community similarity for all facets of plant diversity in the roadside plots, and this effect was stronger for common and dominant species at both scales (**Figure 4**).

### Elevational patterns of plant diversity

Regarding our second research question, our findings revealed a differential effect of road disturbance on plant diversity along the elevation gradient. At least three “best” models were obtained for each facet of diversity (**Table 2**) through the model selection process. The model-averaged estimates of plant diversity and their conditional standard errors were listed in **Table S4**. The contributions of random effects to explaining the variabilities in all plant diversity indices were less than those of fixed effects, as determined by calculating the differences between $R_{c}^{2}$ and $R_{m}^{2}$ (**Table 2**).

**Table 2:** The entire set of plausible models (ΔAICc< 2) for plant diversity indices (taxonomic [^q^TD], functional [^q^FD], phylogenetic [^q^PD]) based on AICc model selection.

Diversity indices	Parameters	AICc	ΔAICc	weight	$R_{m}^{2}$	$R_{c}^{2}$	$R_{c}^{2}-R_{m}^{2}$
**^0^TD**	E+E^2^+R+E*E^2^	**-54.78**	**0.00**	**0.38**	**0.4876**	**0.7147**	**0.2271**
	E+E^2^+R+E*E^2^+E^2^*R	-53.37	1.42	0.19	0.4916	0.7173	0.2257
	E+E^2^+R+E*E^2^+E*R	-53.21	1.57	0.17	0.4907	0.7160	0.2253
**^1^TD**	**E+R**	**-32.40**	**0.00**	**0.27**	**0.3238**	**0.4826**	**0.1588**
	E+E^2^+R	-32.11	0.29	0.23	0.3422	0.4817	0.1395
	E+E^2^+R+E*E^2^	-31.65	0.75	0.19	0.3592	0.4924	0.1332
**^2^TD**	**E+R**	**-22.62**	**0.00**	**0.32**	**0.2646**	**0.4760**	**0.2114**
	E^2^+R	-21.58	1.04	0.19	0.2500	0.4769	0.2269
	E+E^2^+R	-21.19	1.43	0.15	0.2724	0.4779	0.2055
**^0^FD**	E+E^2^+R+E*E^2^	**-54.62**	**0.00**	**0.42**	**0.4608**	**0.6881**	**0.2273**
	E+E^2^+R+E*E^2^+E^2^*R	-53.14	1.48	0.20	0.4644	0.6907	0.2263
	E+E^2^+R+E*E^2^+E*R	-52.97	1.65	0.18	0.4633	0.6891	0.2258
**^1^FD**	E+E^2^+R+E*E^2^	**-32.40**	**0.00**	**0.24**	**0.3717**	**0.4797**	**0.1080**
	E+R	-32.37	0.03	0.24	0.3326	0.4715	0.1389
	E+E^2^+R	-32.28	0.12	0.23	0.3507	0.4745	0.1238
**^2^FD**	**E+R**	**-32.09**	**0.00**	**0.33**	**0.3022**	**0.4671**	**0.1649**
	E+E^2^+R	-30.92	1.16	0.18	0.3079	0.4677	0.1598
	E^2^+R	-30.73	1.36	0.17	0.2872	0.4688	0.1816
**^0^PD**	**E+E^2^+R**	**-37.66**	**0.00**	**0.27**	**0.3685**	**0.4843**	**0.1158**
	E+E^2^+R+E*E^2^	-36.59	1.06	0.16	0.3780	0.4932	0.1152
	E+E^2^+R+E*R+E^2^*R	-36.29	1.36	0.14	0.3876	0.5117	0.1241
**^1^PD**	E+R+E*R	**-81.30**	**0.00**	**0.25**	**0.4554**	**0.4963**	**0.0409**
	E^2^+R+E^2^*R	-81.29	0.01	0.25	0.4543	0.5068	0.0525
	E+E^2^+R+E^2^*R	-80.77	0.53	0.19	0.4654	0.5129	0.0475
	E+E^2^+R+E*R	-79.93	1.37	0.12	0.4600	0.5018	0.0418
**^2^PD**	E+E^2^+R+E*R	**-115.20**	**0.00**	**0.34**	**0.4360**	**0.4482**	**0.0122**
	E+E^2^+R+E^2^*R	-113.88	1.31	0.17	0.4265	0.4301	0.0036
	E+E^2^+R+E*E^2^+E*R	-113.41	1.78	0.14	0.4383	0.4512	0.0129
	E+R+E*R	-113.35	1.84	0.13	0.4062	0.4415	0.0353

ΔAICc and weight correspond to AICc differences and Akaike weights, respectively. Best models are chosen among all models with DAICc<2 using the AICc criteria. Random effects include factor road and site. $R_{m}^{2}$ : Marginal R^2^; $R_{c}^{2}$ : Conditional R^2^. E, Elevation gradient; R, Road disturbance or not. Items in bold represent the model with the smallest AIC.

**Figure 5:**
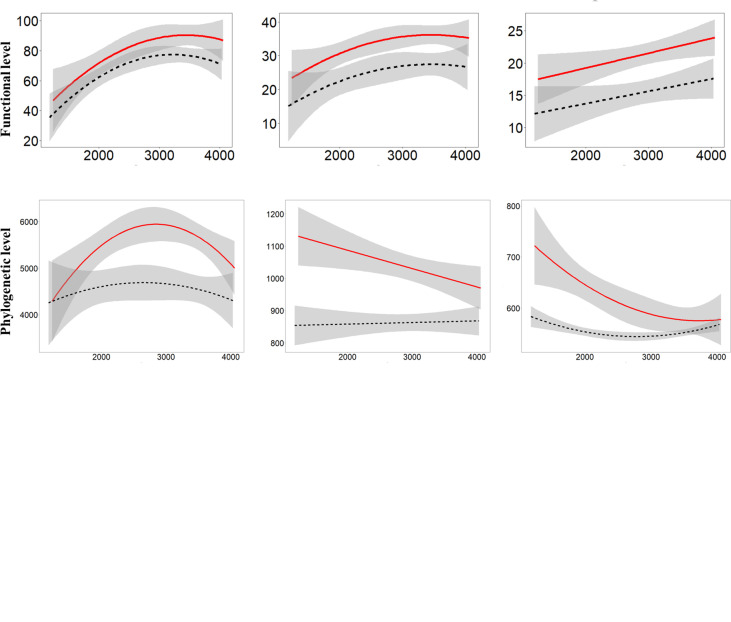
Model predictions (lines) with 95% confidence intervals for each q order (q = [0, 1, 2]) and plant diversity (taxonomic, functional and phylogenetic levels) along elevation gradient. Red solid lines represent predictions in interior plots; black doted lines are used for predictions in the roadside plots.

Most of the best models included the interaction between the plot type and the elevation and/or its quadratic term (**Table 2**), especially when modeling the phylogenetic diversity index. By comparing the plant diversity between the roadside and interior plots, in terms of the taxonomic and functional diversity indices, the negative effects of roads were greater at moderate and high elevations (>3500 m a.s.l) than at low elevations, while the effects of roads on phylogenetic diversity were greater at low and moderate elevations (<2800 m a.s.l) than at high elevations (**Figure 5**).

## Discussion

We investigated the taxonomic, functional and phylogenetic responses of dominant, common and rare species to mountain roads, accounting for shifts in the magnitude of road effects along an elevation gradient. Our results show that dominant species had greater changes to road disturbances than common and rare species at the taxonomic and functional levels, whereas the opposite was true at the phylogenetic level. Furthermore, the greatest effect of roads was reflected in mid- and high-elevation areas at taxonomic and functional levels, while at the phylogenetic level, the greatest road impacts occurred in low- and mid-elevation areas. This study provides fundamental information regarding the effects of roads on plant diversity and may help to forecast future community compositions in response to new road construction in similar areas.

### Roadside communities favor and exclude some species, functional traits and phylogenetic clades

The environmental heterogeneities induced by road construction may act at different spatial scales, thus modifying the compositions of mountain plant communities (Haider et al., 2018; Neher et al., 2013). We found that 14.62% of the total number of species grew exclusively in roadside communities; this finding confirmed that roads act as corridors for the dispersal of plant species (Lembrechts et al., 2017). Most species that appeared only in roadside plots were annual/perennial species that underwent early flowering, had small LDMCs, low SLA values, and anemo- and epizoochory dispersal modes, such as *Cirsium arvense*, *Ajuga ciliate*, *Oxalis corniculata*, *Trifolium pratense*, *and Erigeron acris*. These species are capable of rapid resource uptake, growth and tissue turnover, allowing them to adapt to the often-disturbed and nutrient-rich environments along roads (Balázs et al., 2016; Craven et al., 2018).

In addition, analysis of CWMs of all functional traits revealed no clear difference in functional composition between roadside and interior communities (**Figure 3**). This result is not surprising since abundance-based metrics attenuate the role of rare species in determining overall functional traits (Norden et al., 2017). At the same time, roads drove the absence of species that were present in interior plots. Most of the species that were absent in roadside plots were gymnosperms, such as *Abies fargesii* var. *faxoniana*, *Picea brachytyla*, *Picea likiangensis* var. *rubescens*, *Pinus thunbergii*, and ferns with relatively high SLA values, such as *Adiantum pedatum* and *Athyrium costulalisorum*. The gymnosperm and fern clades are older (Jin and Qian, 2019) than the Asteraceae species favored in the roadside communities (Alexander et al., 2016), suggesting that the changes in functional traits in this study can be attributed to the position of the species in a phylogeny (Munguía-Rosas et al., 2014).

### Mountain roads reduce three facets of plant diversity, but by different magnitudes

A total of 32 nonnative species were found in this survey, including 4 invasive species (Li et al., 2022). Nonnative species that disperse *via* roads often have more similar functional traits (McDougall et al., 2018), so their introduction would lead to a certain decline in the functional diversity of roadside communities. However, considering the relatively small proportion of nonnative species richness in the study area, we believe that the high radiation, high nutrient and high disturbance environment of the roadside community (Johnston and Johnston, 2004; Delgado et al., 2007; De Pauw et al., 2021) led to the disappearance of many native species from the roadside plots, which was the main reason for the decrease of functional diversity. In other words, the communities formed following road construction have lower functional diversity levels than other communities because the environmental filters imposed by the construction of roads select species that are able to cope with these filters and excluded some species with unique functional traits (Laliberté et al., 2014; Li et al., 2018). Some of the older lineages present in the interior communities (such as ferns and gymnosperms) were absent from the roadside communities, thus resulting in the decreased phylogenetic diversity measured in the roadside plots (Arroyo-Rodríguez et al., 2012; Munguía-Rosas et al., 2014; De Pauw et al., 2021).

Furthermore, the effect of roads on the three facets of plant diversity can be explained not only by the decreased species richness but also by the changes that occur in species abundance. Although roads affect overall plant diversity, the multi-comparison of these plant diversity facets showed that road disturbances excluded rare species and reduced the abundance of common and dominant species (**Figure 3**). Thus, our results suggest that roadside plant communities may be more homogenization in the three facets of plant diversity than interior communities. This is consistent with previous findings regarding species richness in mountainous areas (Haider et al., 2018; Li et al., 2022). We detected those changes in taxonomic diversity would exceed changes in functional and phylogenetic diversity, signaling functional and phylogenetic resilience (Jarzyna and Jetz, 2018).

The use of phylogenetic and functional diversity versus taxonomic diversity provides different, complementary information on community assembly mechanisms in plant communities across the Qionglai mountain range. Phylogenetic niche conservatism holds that that closely related species tend to be ecologically similar (Losos, 2008; Wiens et al., 2010). However, phylogenetic niche conservatism was not common in the present study, suggesting that the plant diversity metrics used in the study were decoupled from one another (Grigoropoulou et al., 2022). The results of the correlation analysis also confirmed this result (**Figure S2**). The observed mismatches between phylogenetic and functional diversity in the study might have also been due to nonrandom filtering of species with unfit traits (Hacala et al., 2021; Monge-González et al., 2021). On the other hand, the taxonomic and functional plant diversity indices showed relatively strong positive correlations, suggesting that the community assemblages of mountain plants are ruled by functional differentiation (Freitas et al., 2021; Vega-Álvarez et al., 2019). We argue that roads in the Qionglai mountain range limit the fitness of some species and expand the range of distribution of other species due to the combination of direct disturbances and indirect microhabitat amelioration.

### Mismatched effects of roads on taxonomic, functional and phylogenetic diversity along the elevation gradient

Here, we describe the differential shifts in taxonomic, functional and phylogenetic diversity along an elevation gradient due to roads in the Qionglai mountain range. These results are congruent with previous studies in which increasing taxonomic (Zhang et al., 2009; Li et al., 2022), functional (Zhang et al., 2014) and phylogenetic (Manish and Pandit, 2018; Zu et al., 2019) diversity levels were reported along elevation gradients and can be explained by moderate elevations being the center region for repeated plant migrations between low and high elevations in the alternating processes of glaciation and deglaciation that occurred along the elevation gradient during the Quaternary (Zhong et al., 1979; Zhang et al., 2009). Thus, plants had repeated opportunities to come into contact, hybridize, and become isolated, and for different populations of the same species to become physically separated and genetically divergent, as they moved into new, isolated niches (Liu et al., 2006; Zu et al., 2019).

To adapt to the complex hydrothermal conditions in mid-elevation regions, plants have also evolved diverse functional properties (Dıíaz and Cabido, 2001). However, the abundant species at higher elevations belonged to specific clades, and traits related to harsh environmental filtering at higher elevation evolved independently of phylogeny (Kitagawa et al., 2018). Species that are able to overcome harsh environments and are common/dominant at high elevations have closer evolutionary distances, thus resulting in the relatively low phylogenetic diversity levels of common and dominant species at high elevations (Webb et al., 2002; Qian, 2014; Kitagawa et al., 2018).

When simultaneously considering the effects of roads and elevation gradients, we revealed that the generally negative effects of roads on the three plant diversity facets were more pronounced at moderate and high elevations than at low elevations for taxonomic and functional diversity. In contrast, for phylogenetic diversity, the effects of mountain roads were smaller at high elevations, especially for common and dominant species. The impacts of roads were greater in places with greater diversity, likely because these places had more floral taxa, functional traits and phylogenetic clades that were not adapted to roadside disturbance (Zu et al., 2019). Therefore, planning new roads in a place with large numbers of plant species with unique traits and phylogenetic clades should be a last-resort decision.

## Conclusions and implications for management and conservation

Our studies fill gaps in knowledge regarding the coordination of biodiversity changes in functional, taxonomic and phylogenetic facets of plant communities in response to mountain roads. The results show that mountain roads reduced plant diversity by reducing the abundance of individuals in the community and, to a lesser extent, reducing species richness. Furthermore, we found that the responses of taxonomic and functional diversity to mountain roads were consistent, but the responses of phylogenetic diversity to mountain roads were inconsistent with those of the two other facets of plant diversity. Overall, the multiple facets of plant diversity decrease due to the presence of mountain roads, and that such decreases are not necessarily synchronous or equal along the elevation gradient.

These findings highlight that the importance of considering the multiple facets of plant diversity to understand the effects of roads on plant communities to help management agencies develop effective and rational protection management measures for the national park, since attempts to maximize taxonomic or functional diversity may inadvertently lead to the erosion of phylogenetic diversity. Furthermore, the extent to which montane plant communities may be susceptible to roads depending on their current position along the elevation gradient. When road construction in this area is unavoidable, the road construction plan can be guided according to the facets of diversity with the greatest loss caused by roads, combined with the elevation of the plant community.

## Data availability statement

The original contributions presented in the study are included in the article/**Supplementary Material**. Further inquiries can be directed to the corresponding authors.

## Author contributions

HL: Conceptualization, Methodology, Investigation, Writing-original draft, Writing-review & editing. PL: Writing-review & editing, Funding acquisition. HY: Writing-review & editing. CL, WX, HJ, YC and YH: collected the trait data and conducted the fieldwork. All authors contributed to the article and approved the submitted version.

## Funding

This work was supported by the Demonstration of Monitoring and Protection of Important Species Habitat (grant number 2016YFC0503305); Key Technology and Demonstration for Biodiversity Conservation in Giant Panda National Park (grant number 2018SZDX0036); and the Biodiversity Survey and Assessment Project of the Ministry of Ecology and Environment, People’s Republic of China (grant number 2019HJ2096001006).

## Acknowledgments

Thanks to Dr. Andre F. Clewell for his insightful and helpful comments and edits on this manuscript.

## Conflict of interest

The authors declare that the research was conducted in the absence of any commercial or financial relationships that could be construed as a potential conflict of interest.

## Publisher’s note

All claims expressed in this article are solely those of the authors and do not necessarily represent those of their affiliated organizations, or those of the publisher, the editors and the reviewers. Any product that may be evaluated in this article, or claim that may be made by its manufacturer, is not guaranteed or endorsed by the publisher.
