# Supplementary material for: Assessing the effect of roads on mountain plant diversity beyond species richness
Source: Front Plant Sci. 2022 Sep 26;13:985673. doi: 10.3389/fpls.2022.985673 (PMC9549253; doi:10.3389/fpls.2022.985673)

Supporting information for

Assessing the effect of roads on mountain plant diversity beyond species richness

Table S1. Species names, species abbreviations of species whose abundance is more than 0.1% of the total species abundance.

Species	Species.abbr	GENUS	FAMILY
Abies fabri (Mast.) Craib	Abi.fab	Abies	Pinaceae
Achudemia japonica Maxim.	Ach.jap	Achudemia	Urticaceae
Actaea cimicifuga L.	Act.cim	Actaea	Ranunculaceae
Adenophora capillaris Hemsl.	Ade.cap	Adenophora	Campanulaceae
Agrostis clavate Trin.	Agr.cla	Agrostis	Poaceae
Agrostis pilosula Trin.	Agro.pil	Agrostis	Poaceae
Aletris spicata (Thunb.) Franch.	Ale.spi	Aletris	Nartheciaceae
Anaphalis aureopunctata Lingelsh. & Borza	Ana.aur	Anaphalis	Asteraceae
Anaphalis nepalensis (Spreng.) Hand. - Mazz.	Ana.nep	Anaphalis	Asteraceae
Anemone exigua Maxim.	Ane.exi	Anemone	Ranunculaceae
Anemone hupehensis (É.Lemoine) É.Lemoine	Ane.hup	Anemone	Ranunculaceae

**Species**	**Species.abbr**	**GENUS**	**FAMILY**
Anemone obtusiloba D.Don	Ane.obt	Anemone	Ranunculaceae
Anemone rivularis Buch. -Ham. ex DC.	Ane.riv	Anemone	Ranunculaceae
Artemisia argyi H.Lév. & Vaniot	Art.arg	Artemisia	Asteraceae
Artemisia lancea Vaniot	Art.lan	Artemisia	Asteraceae
Arthraxon hispidus (Thunb.) Makino	Art.his	Arthraxon	Poaceae
Aster ageratoides Turcz.	Ast.age	Aster	Asteraceae
Aster albescens (DC.) Wall. ex Hand. -Mazz.	Ast.alb	Aster	Asteraceae
Aster alpinus L.	Ast.alp	Aster	Asteraceae
Athyrium schizochlamys (Ching) K. Iwats.	Ath.sch	Athyrium	Athyriaceae
Beckwithia glacialis (L.) Á.Löve & D.Löve	Bec.gla	Beckwithia	Ranunculaceae
Boehmeria martini H.Lév.	Boe.mar	Boehmeria	Urticaceae
Brachypodium sylvaticum (Huds.) P.Beauv.	Bra.syl	Brachypodium	Poaceae
Bromus inermis Leyss.	Bro.ine	Bromus	Poaceae
Buddleja davidii Franch.	Bud.dav	Buddleja	Scrophulariaceae
Calamagrostis scabrescens Griseb.	Cal.sca	Calamagrostis	Poaceae
Caltha palustris L.	Cal.pal	Caltha	Ranunculaceae
Cardamine impatiens L.	Car.imp	Cardamine	Brassicaceae

Species	Species.abbr	GENUS	FAMILY
Cardamine purpurascens (O.E.Schulz) Al-Shehbaz & al.	Car.pur	Cardamine	Brassicaceae
Carex doniana Spreng.	Car.don	Carex	Cyperaceae
Carex obscura var. brachycarpa C.B.Clarke	Car.obs	Carex	Cyperaceae
Carex scolopendriformis F.T.Wang & Tang ex P.C.Li	Car.sco	Carex	Cyperaceae
Carpesium lipskyi C.Winkl.	Car.lip	Carpesium	Asteraceae
Cerastium furcatum Cham. & Schltdl.	Cer.fur	Cerastium	Caryophyllaceae
Cerastium glomeratum Thuill.	Cer.glo	Cerastium	Caryophyllaceae
Chrysanthemum przewalskii (Poljakov) H.Ohashi & Yonek.	Chr.prz	Chrysanthemum_	Asteraceae
Circaea cordata Royle	Cir.cor	Circaea	Onagraceae
Circaeaster agrestis Maxim.	Cir.agr	Circaeaster	Circaeasteraceae
Cirsium handelii Petr. ex Hand. -Mazz.	Cir.han	Cirsium	Asteraceae
Cirsium periacanthaceum C.Shih	Cir.per	Cirsium	Asteraceae
Clinopodium polycephalum (Vaniot) C.Y.Wu & S.J.Hsuan	Cli.pol	Clinopodium	Lamiaceae
Clinopodium repens (Buch. -Ham. ex D.Don) Benth.	Cli.rep	Clinopodium	Lamiaceae

Species	Species.abbr	GENUS	FAMILY
Comastoma pulmonarium (Turcz.) Toyok.	Com.pul	Comastoma	Gentianaceae
Corydalis curviflora Maxim. ex Hemsl.	Cor.cur	Corydalis	Papaveraceae
Corydalis pseudobarbisepala Fedde	Cor.pse	Corydalis	Papaveraceae
Cyananthus hookeri C.B.Clarke	Cya.hoo	Cyananthus	Campanulaceae
Cyananthus macrocalyx Franch.	Cya.mac	Cyananthus	Campanulaceae
Dactylis glomerata L.	Dac.glo	Dactylis	Poaceae
Deschampsia cespitosa (L.) P.Beauv.	Des.ces	Deschampsia	Poaceae
Disporum longistylum (H.Lév. & Vaniot) H.Hara	Dis.lon	Disporum	Colchicaceae
Dryopteris transmorrisonensis (Hayata) Hayata	Dry.tra	Dryopteris	Dryopteridaceae
Duchesnea indica (Jacks.) Focke	Duc.ind	Duchesnea	Rosaceae
Elaeagnus rhamnoides (L.) A.Nelson	Ela.rha	Elaeagnus	Elaeagnaceae
Elatostema obtusum Wedd.	Ela.obt	Elatostema	Urticaceae
Elsholtzia ciliate (Thunb.) Hyl.	Els.cil	Elsholtzia	Lamiaceae
Elsholtzia feddei H.Lév.	Els.fed	Elsholtzia	Lamiaceae
Elymus nutans Griseb.	Ely.nut	Elymus	Poaceae
Elymus tangutorum (Nevski) Hand. -Mazz.	Ely.tan	Elymus	Poaceae
Epilobium hirsutum L.	Epi.hir	Epilobium	Onagraceae
Epilobium platystigmatosum C.B.Rob.	Epi.pla	Epilobium	Onagraceae

Species	Species.abbr	GENUS	FAMILY
Epilobium sikkimense Hausskn.	Epi.sik	Epilobium	Onagraceae
Epilobium wallichianum Hausskn.	Epi.wal	Epilobium	Onagraceae
Eragrostis nigra Nees ex Steud.	Era.nig	Eragrostis	Poaceae
Euphorbia micractina Boiss.	Eup.mic	Euphorbia	Euphorbiaceae
Euphrasia pectinate Ten.	Eup.pec	Euphrasia	Orobanchaceae
Festuca fascinate S.L.Lu	Fes.fas	Festuca	Poaceae
Festuca leptopogon Stapf	Fes.lep	Festuca	Poaceae
Fragaria moupinensis (Franch.) Cardot	Fra.mou	Fragaria	Rosaceae
Fragaria orientalis Losinsk.	Fra.ori	Fragaria	Rosaceae
Fragaria pentaphylla Losinsk.	Fra.pen	Fragaria	Rosaceae
Galinsoga parviflora Cav.	Gal.par	Galinsoga	Asteraceae
Galium hoffmeisteri (Klotzsch) Ehrend. & Schönb.-Tem. ex R.R.Mill	Gal.hof	Galium	Rubiaceae
Galium spurium L.	Gal.spu	Galium	Rubiaceae
Gentiana arethusae subsp. delicatula (C.Marquand) Halda	Gen.are	Gentiana	Gentianaceae
Gentiana hexaphylla Maxim. ex Kusn.	Gen.hex	Gentiana	Gentianaceae
Gentianopsis paludosa (Hook.f.) Ma	Gen.pal	Gentianopsis	Gentianaceae
Geranium nepalense Sweet	Ger.nep	Geranium	Geraniaceae
Geranium pylzowianum Maxim.	Ger.pyl	Geranium	Geraniaceae

Species	Species.abbr	GENUS	FAMILY
Geranium refractum Edgew. & Hook.f.	Ger.ref	Geranium	Geraniaceae
Geum aleppicum Jacq.	Geu.ale	Geum	Rosaceae
Halenia elliptica D.Don	Hal.ell	Halenia	Gentianaceae
Hedysarum sikkimense Baker	Hed.sik	Hedysarum	Fabaceae
Hydrangea strigosa Rehder	Hyd.str	Hydrangea	Hydrangeaceae
Impatiens quadriloba K.M.Liu & Y.L.Xiang	Imp.qua	Impatiens	Balsaminaceae
Impatiens undulata Y.L. Chen & Y.Q. Lu	Imp.und	Impatiens	Balsaminaceae
Isolepis setacea (L.) R.Br.	Iso.set	Isolepis	Cyperaceae
Juncus allioides Franch.	Jun.all	Juncus	Juncaceae
Juncus prismatocarpus R.Br.	Jun.pri	Juncus	Juncaceae
Laportea bulbifera (Siebold & Zucc.) Wedd.	Lap.bul	Laportea	Urticaceae
Leontopodium calocephalum (Franch.) Beauverd	Leo.cal	Leontopodium	Asteraceae
Leontopodium wilsonii Beauverd	Leo.wil	Leontopodium	Asteraceae
Ligularia fischeri (Ledeb.) Turcz.	Lig.fis	Ligularia	Asteraceae
Ligularia nelumbifolia (Bureau & Franch.) Hand. -Mazz.	Lig.nel	Ligularia	Asteraceae

Species	Species.abbr	GENUS	FAMILY
Ligularia sagitta (Maxim.) Mattf. ex Rehder & Kobuski	Lig.sag	Ligularia	Asteraceae
Ligusticum hispidum (Franch.) H. Wolff ex Hand. -Mazz.	Lig.his	Ligusticum	Apiaceae
Lilium duchartrei Franch.	Lil.duc	Lilium	Liliaceae
Lolium perenne L.	Lol.per	Lolium	Poaceae
Lomatogonium carinthiacum (Wulfen) A.Braun	Lom.car	Lomatogonium	Gentianaceae
Lotus corniculatus L.	Lot.cor	Lotus	Fabaceae
Matteuccia struthiopteris (L.) Tod.	Mat.str	Matteuccia	Onocleaceae
Medicago lupulina L.	Med.lup	Medicago	Fabaceae
Medicago polymorpha L.	Med.pol	Medicago	Fabaceae
Medicago sativa L.	Med.sat	Medicago	Fabaceae
Microula involucriformis W.T. Wang	Mic.inv	Microula	Boraginaceae
Muhlenbergia japonica Steud.	Muh.jap	Muhlenbergia	Poaceae
Nabalus tatarinowii (Maxim.) Nakai	Nab.tat	Nabalus	Asteraceae
Notopterygium franchetii H.Boissieu	Not.fra	Notopterygium	Apiaceae
Ophiopogon bodinieri H.Lév.	Oph.bod	Ophiopogon	Asparagaceae
Oxalis griffithii Edgew. & Hook. f.	Oxa.gri	Oxalis	Oxalidaceae
Parasenecio latipes (Franch.) Y.L.Chen	Par.lat	Parasenecio	Asteraceae

Species	Species.abbr	GENUS	FAMILY
Parasenecio palmatisectus (Jeffrey) Y.L.Chen	Par.pal	Parasenecio	Asteraceae
Parasenecio roborowskii (Maxim.) Y.L.Chen	Par.rob	Parasenecio	Asteraceae
Parathelypteris glanduligera (Kunze) Ching	Par.gla	Parathelypteris	Thelypteridaceae
Parnassia brevistyla (Brieger) Hand. -Mazz.	Par.bre	Parnassia	Celastraceae
Pedicularis davidii Franch.	Ped.dav	Pedicularis	Orobanchaceae
Pedicularis torta Maxim.	Ped.tor	Pedicularis	Orobanchaceae
Persicaria nepalensis (Meisn.) Miyabe	Per.nep	Persicaria	Polygonaceae
Persicaria vivipara (L.) Ronse Decr.	Per.viv	Persicaria	Polygonaceae
Petasites tricholobus Franch.	Pet.tri	Petasites	Asteraceae
Phleum alpinum L.	Phl.alp	Phleum	Poaceae
Picris hieracioides Sibth. & Sm.	Pic.hie	Picris	Asteraceae
Pilea pumila (L.) A. Gray	Pil.pum	Pilea	Urticaceae
Pimpinella rhomboidei Diels	Pim.rho	Pimpinella	Apiaceae
Plantago asiatica L.	Pla.asi	Plantago	Plantaginaceae
Poa infirma Kunth	Poa.inf	Poa	Poaceae
Poa nemoralis L.	Poa.nem	Poa	Poaceae

**Species**	**Species.abbr**	**GENUS**	**FAMILY**
Poa szechuensis Rendle	Poa.sze	Poa	Poaceae
Polygonatum curvistylum Hua	Pol.cur	Polygonatum	Asparagaceae
Polygonum cyanandrum Diels	Pol.cya	Polygonum	Polygonaceae
Polygonum sparsipilosum A.J.Li	Pol.spa	Polygonum	Polygonaceae
Potentilla anserina L.	Pot.ans	Potentilla	Rosaceae
Potentilla leuconota D.Don	Pot.leu	Potentilla	Rosaceae
Potentilla potaninii Th.Wolf	Pot.pot	Potentilla	Rosaceae
Primula deflexa Duthie	Pri.def	Primula	Primulaceae
Primula optata Farrer	Pri.opt	Primula	Primulaceae
Primula palmata Hand. -Mazz.	Pri.pal	Primula	Primulaceae
Primula sikkimensis Hook.	Pri.sik	Primula	Primulaceae
Prunella vulgaris L.	Pru.vul	Prunella	Lamiaceae
Ranunculus brotherusii var. tanguticus (Maxim.) Tamura	Ran.bro	Ranunculus	Ranunculaceae
Ranunculus japonicus Thunb.	Ran.jap	Ranunculus	Ranunculaceae
Rhodiola kirilowii (Regel) Maxim.	Rho.kir	Rhodiola	Crassulaceae
Rodgersia aesculifolia Batalin	Rod.aes	Rodgersia	Saxifragaceae
Rubus fockeanus Kurz	Rub.foc	Rubus	Rosaceae
Rubus lasiostylus Focke	Rub.las	Rubus	Rosaceae
Rubus mesogaeus Focke	Rub.mes	Rubus	Rosaceae

Species	Species.abbr	GENUS	FAMILY
Rubus xanthocarpus Bureau & Franch.	Rub.xan	Rubus	Rosaceae
Rumex acetosa L.	Rum.ace	Rumex	Polygonaceae
Rumex dentatus L.	Rum.den	Rumex	Polygonaceae
Salix erioclada H. Lév. & Vaniot	Sal.eri	Salix	Salicaceae
Salvia przewalskii Maxim.	Sal.prz	Salvia	Lamiaceae
Saussurea leontodontoides (DC.) Sch.Bip.	Sau.leo	Saussurea	Asteraceae
Saussurea salicifolia (L.) DC.	Sau.sal	Saussurea	Asteraceae
Saussurea tatsienensis Bureau & Franch.	Sau.tat	Saussurea	Asteraceae
Saussurea woodiana Hemsl.	Sau.woo	Saussurea	Asteraceae
Saxifraga egregia Engl.	Sax.egr	Saxifraga	Saxifragaceae
Saxifraga stellariifolia Franch.	Sax.ste	Saxifraga	Saxifragaceae
Sedum rosei Raym. -Hamet	Sed.ros	Sedum	Crassulaceae
Sedum wenchuanense S.H. Fu	Sed.wen	Sedum	Crassulaceae
Selaginella braunii Baker	Sel.bra	Selaginella	Selaginellaceae
Sinacalia davidii (Franch.) H.Koyama	Sin.dav	Sinacalia	Asteraceae
Sinacalia tangutica (Maxim.) B.Nord.	Sin.tan	Sinacalia	Asteraceae
Smilax stans Maxim.	Smi.sta	Smilax	Smilacaceae
Stellaria decumbens Edgew.	Ste.dec	Stellaria	Caryophyllaceae
Stellaria media (L.) Vill.	Ste.med	Stellaria	Caryophyllaceae
Taraxacum mongolicum Hand. -Mazz.	Tar.mon	Taraxacum	Asteraceae

Species	Species.abbr	GENUS	FAMILY
Tibetia himalaica (Baker) H.P.Tsui	Tib.him	Tibetia	Fabaceae
Torilis scabra (Thunb.) DC.	Tor.sca	Torilis	Apiaceae
Trifolium repens L.	Tri.rep	Trifolium	Fabaceae
Triplostegia glandulifera Wall. ex DC.	Tri.gla	Triplostegia	Caprifoliaceae
Trisetum clarkei (Hook.f.) R.R.Stewart	Tri.cla	Trisetum	Poaceae
Trollius buddae Schipcz.	Tro.bud	Trollius	Ranunculaceae
Urtica thunbergiana Siebold & Zucc.	Urt.thu	Urtica	Urticaceae
Valeriana jatamansi Jones	Val.jat	Valeriana	Caprifoliaceae
Veronica szechuanica Batalin	Ver.sze	Veronica	Plantaginaceae
Vicia cracca L.	Vic.cra	Vicia	Fabaceae
Vicia unijuga A.Br.	Vic.uni	Vicia	Fabaceae
Viola szetschwanensis W. Becker & H. Boissieu	Vio.sze	Viola	Violaceae

Table S2. Species names, species abbreviations of species whose abundance is less than 0.001% of the total species abundance.

Species	Species.abrr	GENUS	FAMILY
Acer pectinatum subsp. laxiflorum (Pax) A.E.Murray	Ace.lax	Acer	Sapindaceae
Acer pictum Thunb.	Ace.mon	Acer	Sapindaceae
Acer sterculiaceum Wall.	Ace.ste	Acer	Sapindaceae
Aconitum hemsleyanum E.Pritz.	Aco.hem	Aconitum	Ranunculaceae
Ainsliaea latifolia (D. Don) Sch.Bip.	Ain.lat	Ainsliaea	Asteraceae
Ajuga ciliata Bunge	Aju.cil	Ajuga	Lamiaceae
Alangium chinense (Lour.) Harms	Ala.chi	Alangium	Cornaceae
Aletris laxiflora Bureau & Franch.	Ale.lax	Aletris	Nartheciaceae
Ampelopsis glandulosa (Wall.) Momiy.	Amp.gla	Ampelopsis	Vitaceae
Artemisia annua L.	Art.ann	Artemisia	Asteraceae
Athyrium mackinnonii (C. Hope) C. Chr.	Ath.mac	Athyrium	Athyriaceae
Athyrium wangii Ching	Ath.wan	Athyrium	Athyriaceae
Bauhinia corymbosa Roxb.	Bau.cor	Bauhinia	Fabaceae

**Species**	**Species.abrr**	**GENUS**	**FAMILY**
Begonia grandis subsp. sinensis (A.DC.) Irmsch.	Beg.sin	Begonia	Begoniaceae
Berberis multiovula T.S.Ying	Ber.mul	Berberis	Berberidaceae
Boschniakia himalaica Hook.f. & Thomson	Bos.him	Boschniakia	Orobanchaceae
Brassica rapa L.	Bra.chi	Brassica	Brassicaceae
Bromus pectinatus Thunb.	Bro.pec	Bromus	Poaceae
Carduus nutans L.	Car.nut	Carduus	Asteraceae
Carex alopecuroides D.Don ex Tilloch & Taylor	Car.alo	Carex	Cyperaceae
Carex atrofusca subsp. minor (Boott) T.Koyama	Car.min	Carex	Cyperaceae
Carex pumila Thunb.	Car.pum	Carex	Cyperaceae
Catolobus pendulus (L.) Al-Shehbaz	Cat.pen	Catolobus	Brassicaceae
Chamabainia cuspidate Wight	Cha.cus	Chamabainia	Urticaceae
Clematis urophylla Franch.	Cle.uro	Clematis	Ranunculaceae
Cornus controversa Hemsl.	Cor.con	Cornus	Cornaceae
Cornus schindleri Wangerin	Cor.sch	Cornus	Cornaceae
Corydalis davidii Franch.	Cor.dav	Corydalis	Papaveraceae

Species	Species.abrr	GENUS	FAMILY
Cuscuta reflexa Roxb.	Cus.ref	Cuscuta	Convolvulaceae
Cyclogramma flexilis (Christ) Tagawa	Cyc.fle	Cyclogramma	Thelypteridaceae
Cynanchum bungei Decne.	Cyn.bun	Cynanchum	Apocynaceae
Cynanchum otophyllum C.K.Schneid.	Cyn.oto	Cynanchum	Apocynaceae
Cypripedium tibeticum King ex Rolfe	Cyp.tib	Cypripedium	Orchidaceae
Davidia involucrata Baill.	Dav.inv	Davidia	Nyssaceae
Decaisnea insignis (Griff.) Hook.f. & Thomson	Dec.ins	Decaisnea	Lardizabalaceae
Delphinium trichophorum Franch.	Del.tri	Delphinium	Ranunculaceae
Dendropanax proteus (Champ. ex Benth.) Benth.	Den.pro	Dendropanax	Araliaceae
Dennstaedtia wilfordii (T. Moore) Christ	Den.wil	Dennstaedtia	Dennstaedtiaceae
Deutzia pilosa Rehder	Deu.pil	Deutzia	Hydrangeaceae
Draba subamplexicaulis C.A.Mey.	Dra.sub	Draba	Brassicaceae
Dryopteris barbigera (T. Moore ex Hook.) Kuntze	Dry.bar	Dryopteris	Dryopteridaceae

Species	Species.abrr	GENUS	FAMILY
Duhaldea cappa (Buch. -Ham. ex D.Don) Pruski & Anderb.	Duh.cap	Duhaldea	Asteraceae
Elaeagnus lanceolata Warb.	Ela.lan	Elaeagnus	Elaeagnaceae
Eleutherococcus giraldii (Harms) Nakai	Ele.gir	Eleutherococcus	Araliaceae
Eleutherococcus henryi Oliv.	Ele.hen	Eleutherococcus	Araliaceae
Eleutherococcus leucorrhizus Oliv.	Ele.leu	Eleutherococcus	Araliaceae
Eremochloa ciliaris (L.) Merr.	Ere.cil	Eremochloa	Poaceae
Euonymus hamiltonianus Wall.	Euo.ham	Euonymus	Celastraceae
Eupatorium chinense L.	Eup.chi	Eupatorium	Asteraceae
Eupatorium fortunei Turcz.	Eup.for	Eupatorium	Asteraceae
Fargesia nitida (Mitford) Keng f. ex T.P.Yi	Far.nit	Fargesia	Poaceae
Ficus tikoua Bureau	Fic.tik	Ficus	Moraceae
Gagea serotina (L.) Ker Gawl.	Gag.ser	Gagea	Liliaceae
Gentiana nanobella C.Marquand	Gen.nan	Gentiana	Gentianaceae
Gentiana straminea Maxim.	Gen.str	Gentiana	Gentianaceae
Gentiana suborbisepala C.Marquand	Gen.sub	Gentiana	Gentianaceae
Ginkgo biloba L.	Gin.bil	Ginkgo	Ginkgoaceae

Species	Species.abrr	GENUS	FAMILY
Gymnocarpium jessoense (Koidz.) Koidz.	Gym.jes	Gymnocarpium	Cystopteridaceae
Iris tectorum Maxim.	Iri.tec	Iris	Iridaceae
Juniperus saltuaria Rehder & E.H.Wilson	Jun.sal	Juniperus	Cupressaceae
Leycesteria formosa Wall.	Ley.for	Leycesteria	Caprifoliaceae
Ligustrum lucidum W.T.Aiton	Lig.luc	Ligustrum	Oleaceae
Lonicera rupicola Hook. f. & Thomson	Lon.rup	Lonicera	Caprifoliaceae
Malus pumila Mill.	Mal.pum	Malus	Rosaceae
Osmunda japonica Thunb.	Osm.jap	Osmunda	Osmundaceae
Panax japonicus (T.Nees) C.A.Mey.	Pan.jap	Panax	Araliaceae
Parasenecio profundorum (Dunn) Y.L.Chen	Par.pro	Parasenecio	Asteraceae
Paris polyphylla Sm.	Par.pol	Paris	Melanthiaceae
Peucedanum turgeniifolium H. Wolff	Peu.tur	Peucedanum	Apiaceae
Phytolacca acinosa Roxb.	Phy.aci	Phytolacca	Phytolaccaceae
Pinus thunbergii Parl.	Pin.thu	Pinus	Pinaceae

**Species**	**Species.abrr**	**GENUS**	**FAMILY**
Polystichum makinoi (Tagawa) Tagawa	Pol.mak	Polystichum	Dryopteridaceae
Potentilla fruticosa L.	Pot.fru	Potentilla	Rosaceae
Rhodiola fastigiata (Hook. f. & Thomson) S.H. Fu	Rho.fas	Rhodiola	Crassulaceae
Rhododendron decorum Franch.	Rho.dec	Rhododendron	Ericaceae
Rhododendron faberi subsp. prattii (Franch.) D.F. Chamb.	Rho.pra	Rhododendron	Ericaceae
Rhododendron przewalskii Maxim.	Rho.prz	Rhododendron	Ericaceae
Rosa sericea Wall. ex Lindl.	Ros.ser	Rosa	Rosaceae
Rubia magna P.G.Xiao	Rub.mag	Rubia	Rubiaceae
Rubus biflorus Buch. -Ham. ex Sm.	Rub.bif	Rubus	Rosaceae
Rubus playfairianus Hemsl. ex Focke	Rub.pla	Rubus	Rosaceae
Salix hylonoma C.K. Schneid.	Sal.hyl	Salix	Salicaceae
Satyrium nepalense var. ciliatum (Lindl.) Hook.f.	Sat.cil	Satyrium	Orchidaceae
Schizopepon dioicus Cogn. ex Oliv.	Sch.dio	Schizopepon	Cucurbitaceae
Selaginella vardei H. Lév.	Sel.var	Selaginella	Selaginellaceae

Species	Species.abrr	GENUS	FAMILY
Sibiraea angustata (Rehder) Hand. -Mazz.	Sib.ang	Sibiraea	Rosaceae
Solanum americanum Mill.	Sol.ame	Solanum	Solanaceae
Sonchus oleraceus (L.) L.	Son.ole	Sonchus	Asteraceae
Sorbus prattii Koehne	Sor.pra	Sorbus	Rosaceae
Spiraea henryi Hemsl.	Spi.hen	Spiraea	Rosaceae
Spiraea longigemmis Maxim.	Spi.lon	Spiraea	Rosaceae
Stellaria alaschanica Y.Z. Zhao	Ste.ala	Stellaria	Caryophyllaceae
Stellaria umbellata Turcz.	Ste.umb	Stellaria	Caryophyllaceae
Swertia nervosa (Wall. ex G. Don) C.B. Clarke	Swe.ner	Swertia	Gentianaceae
Thalictrum rostellatum Hook. f. & Thomson	Tha.ros	Thalictrum	Ranunculaceae
Tofieldia thibetica Franch.	Tof.thi	Tofieldia	Tofieldiaceae
Youngia japonica (L.) DC.	You.jap	Youngia	Asteraceae
Zanthoxylum armatum DC.	Zan.arm	Zanthoxylum	Rutaceae

Table S3. Functional traits of plant species and data sources (Flora of China, TRY, measured in the field).

Habit (Fl. China)

annual/biennial, herbaceous perennial, semi-shrub, shrub, tree, vine, or parasitic

Root System (Fl. China)

Tap or fibrous

Leaves (Fl. China)

Texture: membranous, coriaceous, papery, or fleshy

Nitrogen Fixation (TRY, Fl. China)

yes, no

Shade Tolerance (TRY, Fl. China)

Tolerant or intolerant

Heliophile (TRY, Fl. China)

yes, no

Leaf Dry Matter Content (Measured)

numerical data: mg

Special Leaf Area (Measured)

numerical data: m^2/kg

Leaf Thickness (Measured)

numerical data: mm

Principal Reproductive Mode (Fl. China)

vegetative or seed

Initiation of Flowering (Fl. China)

month

Seed Dispersal (TRY, Fl. China)

gravity, wind, or animal

Table S4. Parameter estimates and standard errors from best-fit or averaged linear mixed models on the effects of road and elevation on plant diversity indices (n=76 plots). Random factors: Road (2 levels) and Site (38 levels). SE: Standard Error; SD: Standard Deviation. Note: *** P<0.001; ** P< 0.01; * P< 0.05.

**Taxonomic diversity for *q*=0**			
**Random effects**	**1|Road/Site（SD=0.1221）**		
**Fixed effects**	**Estimate**	**SE**	**t-Value**
(Intercept)	0.198	0.126	1.569
Plot category	-0.143	0.028	-5.106***
Elevation	-0.375	0.907	-0.414
Elevation^2	3.177	1.898	1.674
Elevation*Elevation^2	-2.607	1.181	-2.207*

**Taxonomic diversity for *q*=1**			
**Random effects**	**1|Road/Site** (SD = 0.1633)		
**Fixed effects**	**Estimate**	**SE**	**t-Value**
(Intercept)	0.353	0.065	5.433***
Plot category	-0.202	0.037	-5.394***
Elevation	0.274	0.091	3.009**

Taxonomic diversity for *q*=2			
**Random effects**	**1|Road/Site** (SD = 0.1691)		
**Fixed effects**	**Estimate**	**SE**	**t-Value**
(Intercept)	0.381	0.070	5.425***
Plot category	-0.206	0.039	-5.304***
Elevation	0.208	0.099	2.093*

Functional diversity for *q*=0			
**Random effects**	**1|Road/Site** (SD = 0.1239)		
**Fixed effects**	**Estimate**	**SE**	**t-Value**
(Intercept)	0.226	0.125	1.807
Plot category	-0.146	0.028	-5.128***
Elevation	-0.575	0.896	-0.642
$Elevation^2$	3.470	1.870	1.856
$Elevation*Elevation^2$	-2.770	1.162	-2.384*

Functional diversity for *q*=1			
**Random effects**	**1|Road/Site** (SD = 0.1646)		
**Fixed effects**	**Estimate**	**SE**	**t-Value**
(Intercept)	0.381	0.126	3.018**

Plot category	-0.215	0.038	-5.696***
Elevation	-0.473	0.906	-0.523
Elevation^2	2.409	1.896	1.271
Elevation*Elevation^2	-1.851	1.179	-1.569

**Functional diversity for *q*=2**			
**Random effects**	**1|Road/Site** (SD = 0.1626)		
**Fixed effects**	**Estimate**	**SE**	**t-Value**
(Intercept)	0.359	0.061	5.871***
Plot category	-0.207	0.037	-5.556***
Elevation	0.219	0.087	2.522**

**Phylogenetic diversity for *q*=0**			
**Random effects**	**1|Road/Site** (SD = 0.160)		
**Fixed effects**	**Estimate**	**SE**	**t-Value**
(Intercept)	0.381	0.090	4.219***
Plot category	-0.245	0.037	-6.682***
Elevation	0.941	0.376	2.503*
Elevation^2	-0.842	0.340	-2.479*

Phylogenetic diversity for *q*=1			
**Random effects**	**1|Road/Site** (SD = 0.127)		
**Fixed effects**	**Estimate**	**SE**	**t-Value**
(Intercept)	0.624	0.051	12.249***
Plot category	-0.364	0.069	-5.301***
Elevation	-0.213	0.076	-2.806**
Elevation*Plot category	0.232	0.103	2.243*

Phylogenetic diversity for *q*=2			
**Random effects**	**1|Road/Site** (SD = 0.103)		
**Fixed effects**	**Estimate**	**SE**	**t-Value**
(Intercept)	0.530	0.057	9.287***
Plot category	-0.311	0.056	-5.563***
Elevation	-0.735	0.213	-3.455**
$Elevation^2$	0.384	0.186	2.057*
Elevation*Plot category	0.311	0.084	3.701***

Figure S1. The Hill number-based diversity of vascular plants at two plant communities for increasing order q values (0 to 3): (a) taxonomic level; (b) functional level; and (c) phylogenetic level.

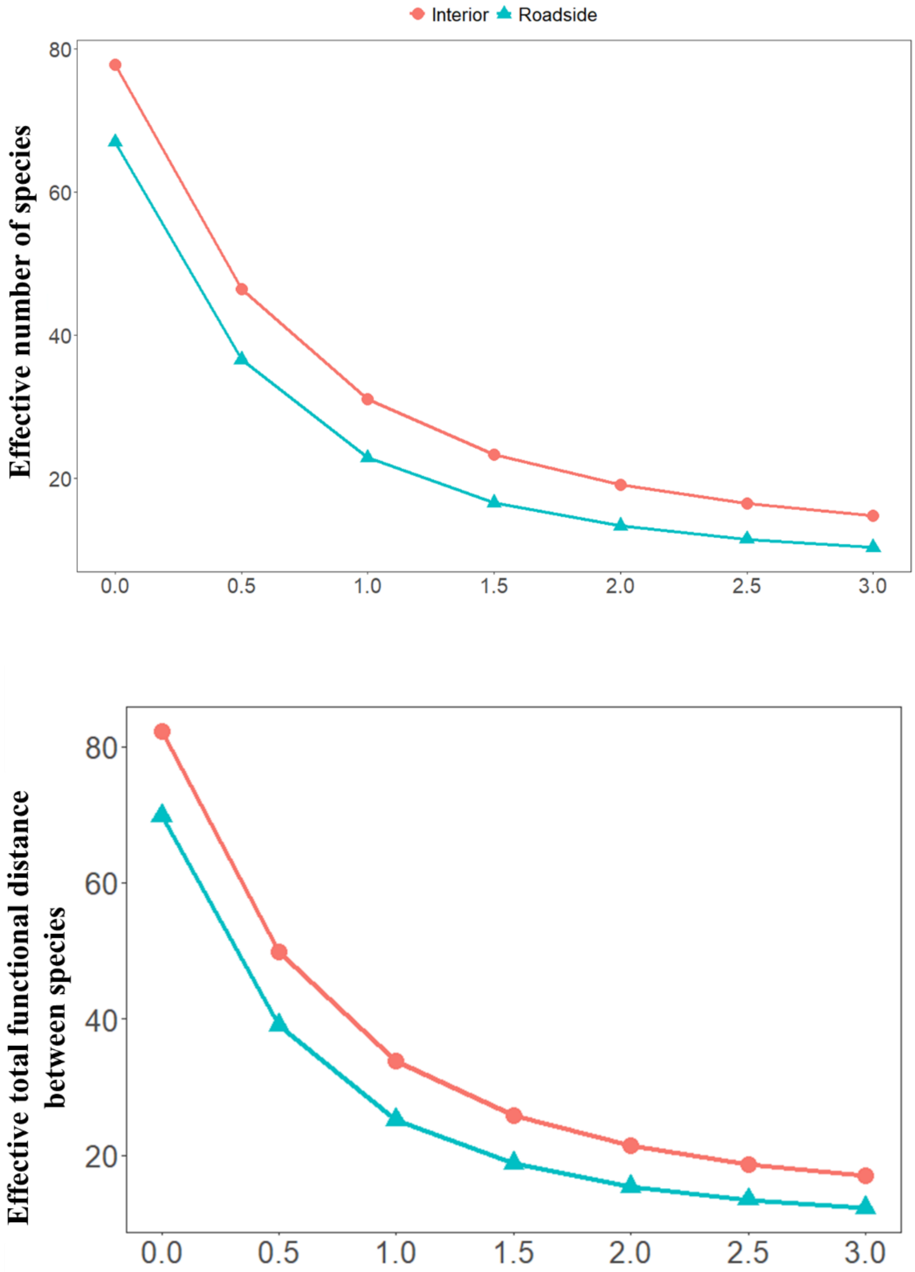

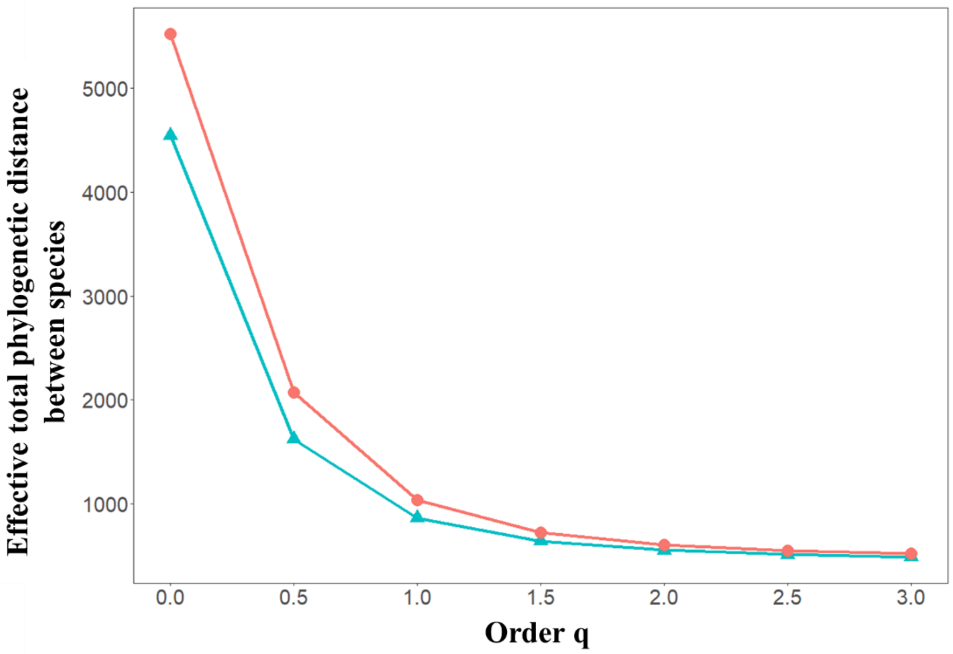

Effective total phylogenetic distance between species
5000
4000
3000
2000
1000
0.0
0.5
1.0
1.5
2.0
2.5
3.0
Order q

Figure S2. Pearson correlations between taxonomic, functional and phylogenetic Hill numbers, for q= [0, 1, 2].

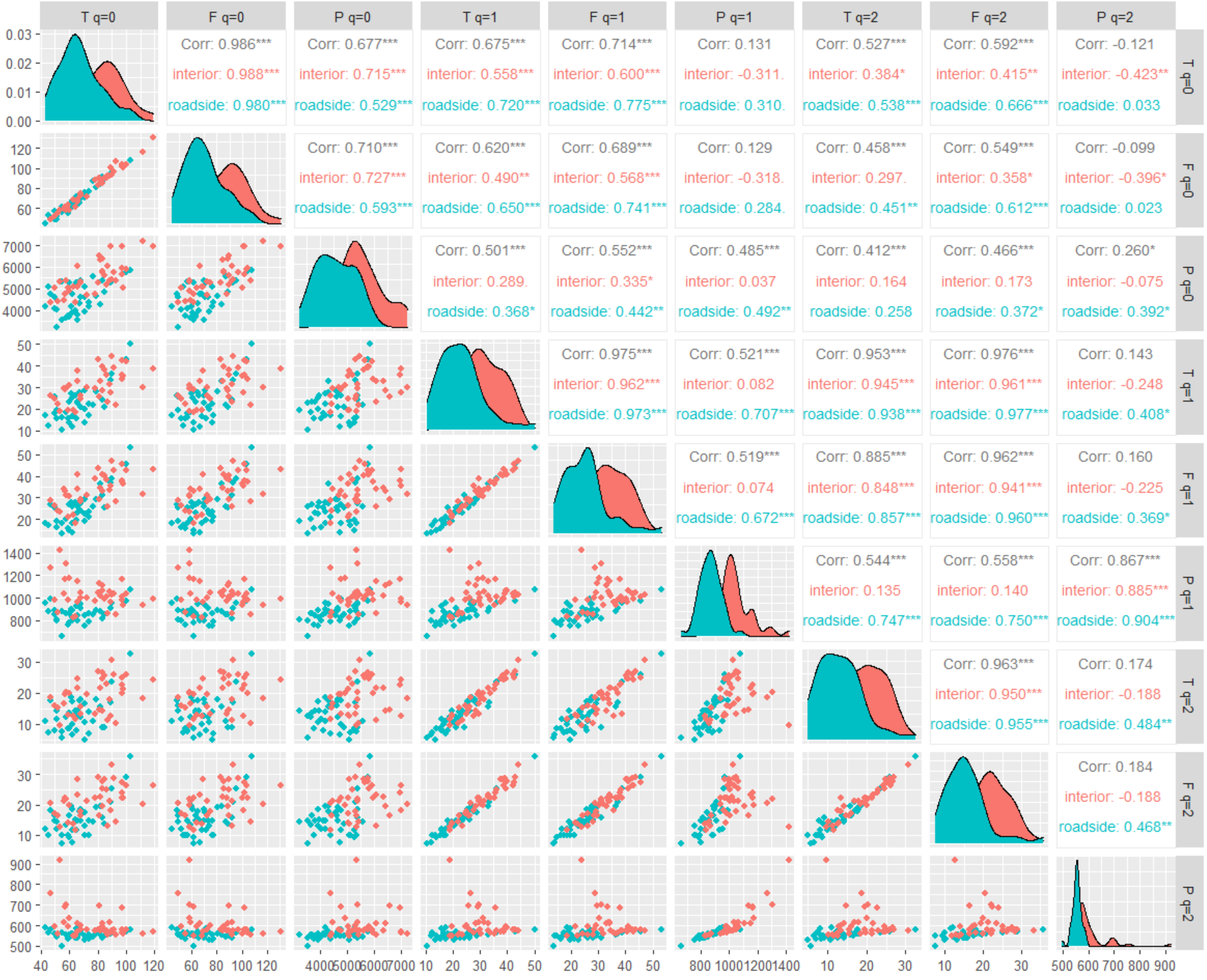

Supplement: Supplementary file 1 [file DataSheet_1.pdf]
